# Supplementary material for: Production efficiency and change characteristics of China’s apple industry in terms of planting scale
Source: PLoS One. 2021 Jul 22;16(7):e0254820. doi: 10.1371/journal.pone.0254820 (PMC8297839; doi:10.1371/journal.pone.0254820)
Supplement: Attachment — Submitted filename: Zhichkin review.docx [file pone.0254820.s002.docx]

Review on PLOS ONE manuscript

**Production Efficiency and Change Characteristics of China’s Apple Industry in Terms of Planting Scale**

**Yu Sun, Zichun Wang,Yonghua Lu, Mingyue Li**

The article is written on a relevant topic. The problem of the location of agricultural production, its efficiency and methods for assessing efficiency is very important. The presence of peculiarities of agricultural production (seasonality, interaction with biological objects, the influence of climate, etc.) require special approaches to assessing the results of production activities, different from industrial or commercial. The problem of apple production in China, the definition of technical equipment and the influence of various factors on it, considered in the article, is relevant not only for the conditions of China, but also for other countries, including Russia. The presence of a large number of small producers, their poor technical equipment, the absence of modern intensive technologies, all this negatively affects the production efficiency.

1. The study presents the results of original research.

The presented study is original and takes into account the specifics of a particular country (China) and its individual regions.

2. Results reported have not been published elsewhere.

There is no information on the republishing of the results.

3. Experiments, statistics, and other analyses are performed to a high technical standard and are described in sufficient detail.

The article uses an extensive statistical apparatus. The approach to the study of the problem is original, which increases its theoretical and practical value. The testing of the hypotheses put forward is multifaceted, the data obtained are confirmed from several sources.

At the same time, there are shortcomings or controversial points in the study. One of the variables of the analyzed model is land area. But nowhere is it specified whether we mean the total area under apple plantations or the area of fruit-bearing orchards. The fact is that there is a significant time gap between the period of setting up orchards and the beginning of their fruiting. And accordingly, the area of young orchards cannot be taken into account when determining the yield of apples. Secondly, there are significant differences between traditional and intensive apple production technologies, therefore, to obtain a more accurate result, they should be considered separately. Thirdly. As practice shows, the main source of improving technical equipment, the introduction of new technologies is large-scale agricultural production, which has large financial and land resources, opportunities for high-quality training of personnel, etc. The authors, however, leave this moment outside the scope of the study. Fourthly, the authors do not provide the initial information used for the calculations, which does not allow checking the correctness of the calculations.

4. Conclusions are presented in an appropriate fashion and are supported by the data.

The conclusions in the main part correspond to the above calculations. The authors creatively use the results obtained and draw, on their basis, detailed conclusions that are of significant practical importance for the conditions of China. The conclusions made are substantiated and consistent.

5. The article is presented in an intelligible fashion and is written in standard English.

The language of writing the article is understandable and meets all professional and linguistic standards of presentation.

6. The research meets all applicable standards for the ethics of experimentation and research integrity.

The presented research fully complies with research ethics standards. No signs of adjustment results found.

7. The article adheres to appropriate reporting guidelines and community standards for data availability.

The article follows the rules for preparing reports. The data used is, in principle, available from the sources indicated in the article. However, it would be clearer if the initial calculation data were given directly in the article.

12/05/2021
