# Supplementary material for: Production efficiency and change characteristics of China’s apple industry in terms of planting scale
Source: PLoS One. 2021 Jul 22;16(7):e0254820. doi: 10.1371/journal.pone.0254820 (PMC8297839; doi:10.1371/journal.pone.0254820)
Supplement: Attachment — Submitted filename: Response to Reviewers.doc [file pone.0254820.s003.doc]

**Reviewer #1:**

Thank you very much. We have updated our list of keywords, replaced the “apple industry” with “apple production,” and reworked the original introduction into a separate section as a literature review to render our manuscript clearer.

Thank you for your valuable advice.

**Reviewer #2:**

Thank you very much. Paper data from China’s official data “The National Agricultural Cost-Benefit Data Compilation,” “The China Agricultural Statistical Yearbook,” and “The National Agricultural Cost-Benefit Data Compilation” in the capital investment costs including technology. There are no climate factors, or factors such as time, organization, and technology. This issue requires a lot of field research. Future research will pay more attention to this.Some necessary natural conditions and other variables can be included in the model for further investigation.

Thank you for your valuable advice.

**Reviewer #3:**

Thank you very much for your valuable advice.

First, as mentioned in the conclusion, the specific contents are as follows. In this study, we used the Malmquist productivity index in the context of the DEA method to assess the industrial efficiency of the seven main apple-producing areas of China and then used a threshold model to analyze the nonlinear characteristics of the change in apple production scale and efficiency in China. The influence of apple planting scale on production efficiency is as follows:

Apple production efficiency in different regions of China showed regional differences and time-series fluctuations. The apple planting scale had a threshold effect, and the impact on apple production efficiency showed a "negative effect–positive effect" trend. Specifically, when the scale of apple planting does not exceed the threshold value of 7.515, the scale hurts apple production efficiency. When the apple planting scale is between 7.515 and 7.691, the effect of the apple planting scale on apple production efficiency becomes a positive effect. When the scale of apple planting exceeds the second threshold value, the effect of scale on apple production efficiency becomes smaller. The impact of rural human capital and local economic development level on apple production efficiency is positively correlated while the impact of natural disasters on apple productivity is negatively correlated.

I regret that my choice of expression caused misunderstanding. All data in this paper are from China Agricultural Statistical Yearbook, the National Agricultural Cost-Benefit Data Compilation, the website of the National Bureau of Statistics, and the China Agricultural Information Network. Other data not included in the statistics have not been used in this paper. The data in this paper are not based on data extrapolated from other years.I adjusted and deleted the ambiguous sentence in the original.

National Bureau of Statistics: <http://www.stats.gov.cn/>

China Agricultural Information Network: http://www.agri.cn/

Thank you for your valuable advice.
